# Neural activity associated with self-reflection

**DOI:** 10.1186/1471-2202-13-52

**Published:** 2012-05-24

**Authors:** Uwe Herwig, Tina Kaffenberger, Caroline Schell, Lutz Jäncke, Annette B Brühl

**Affiliations:** 1 Department for Social and General Psychiatry Zurich West, University Hospital of Psychiatry Zurich, Zurich, Switzerland; 2 Department of Psychiatry and Psychotherapy III, University of Ulm, Ulm, Germany; 3 Department of Neurology, University Hospital Tübingen, Tübingen, Germany; 4 Department of Neuropsychology, Institute of Psychology, University of Zurich, Zurich, Switzerland

## Abstract

**Background:**

Self-referential cognitions are important for self-monitoring and self-regulation. Previous studies have addressed the neural correlates of self-referential processes in response to or related to external stimuli. We here investigated brain activity associated with a short, exclusively mental process of self-reflection in the absence of external stimuli or behavioural requirements. Healthy subjects reflected either on themselves, a personally known or an unknown person during functional magnetic resonance imaging (fMRI). The reflection period was initialized by a cue and followed by photographs of the respective persons (perception of pictures of oneself or the other person).

**Results:**

Self-reflection, compared with reflecting on the other persons and to a major part also compared with perceiving photographs of one-self, was associated with more prominent dorsomedial and lateral prefrontal, insular, anterior and posterior cingulate activations. Whereas some of these areas showed activity in the “other”-conditions as well, self-selective characteristics were revealed in right dorsolateral prefrontal and posterior cingulate cortex for self-reflection; in anterior cingulate cortex for self-perception and in the left inferior parietal lobe for self-reflection and -perception.

**Conclusions:**

Altogether, cingulate, medial and lateral prefrontal, insular and inferior parietal regions show relevance for self-related cognitions, with in part self-specificity in terms of comparison with the known-, unknown- and perception-conditions. Notably, the results are obtained here without behavioural response supporting the reliability of this methodological approach of applying a solely mental intervention. We suggest considering the reported structures when investigating psychopathologically affected self-related processing.

## Background

Humans not only have a neural representation of the external and social world, they also have the ability to represent themselves as coherent human beings and as a self. They can reflect on themselves as a person and they have a neural representation of their own body. Understanding the basis of neural self-representation is not only interesting from a philosophical or scientific point of view but may also have practical implications in psychiatry, for example, in understanding disturbed self-related functions occurring during depression [1].

In the last decade, a growing number of studies has assessed the neural bases of self-related processes using functional neuroimaging methods [2-11] and electrical tomographic techniques [12]. In these studies, brain activity was examined, for example, while viewing photographs of oneself compared to that obtained while viewing photographs of other persons, or while recognizing one’s own face, names, voices or morphed photographs [3,4,13-19]. Other approaches have addressed the relevance of trait adjectives with reference to oneself and compared brain activity to that occurring under control conditions, for example reference of trait adjectives to close friends or others, or have applied more complex self-referential tasks which required responses to external stimuli [2,3,12,20-25]. Self-referential memory [26-28], emotional domains [29,30] and cognitive aspects in the spatial domain such as navigational tasks or perspective-taking tasks [31,32] have also been examined. Self-reference has been further studied in relation to the external world or from the perspective of the self as an object, i.e. from the perspective of a third-person [9,33,34] or relative to certain features of the self, for example individual goals [35], psychological aspects [36] and social issues [20].

Recent meta-analyses of functional neuroimaging studies have confirmed the involvement of certain brain regions in self-referential processes [6,10,37]: medial prefrontal cortex (MPFC), anterior and posterior cingulate cortices (ACC, PCC) and (pre)cuneus, which also reflect the concept of cortical midline areas and self-reference [38]. Further, the lateral and ventromedial prefrontal, medial temporal, and parieto-temporal regions, the insular cortex and other regions were found to be active during self-referential processes [6,9,10,25,37].

From a methodological perspective, all these studies have in common that they examine the neural bases of self-reference in response to external stimuli or in comparison to behavioural tasks potentially involving other cognitive domains. This could make it difficult to assess self-referential activity possible interferences from neural activity related to external cognitions or actions. Certainly, these studies acknowledged the methodological issue of implementing behavioural controls. However, it may be of value to assess whether or not the brain areas reported are also active during a restrictively mental and not behavioural self-referential condition, and whether these areas show more or less specific activity for self-reference than other areas in terms of activation during self-reflection but not during reflection about others or during self-photo perception. In this study, we investigated the neural processes underlying self-reference in the sense of self-reflection, however, compared to other studies without possibly interfering for instance visual or verbal stimuli. The term “self-reflection” comprises at very least processes such as becoming aware of and reflecting on one’s current and past experiences and one’s self-concept, including the self-relevance of trait words [39]. The division between self-reflection, and self-recognition or self-awareness and even self-consciousness is not clear-cut [38,40,41]. Some authors have defined self-reflection as cognitively reflecting on one’s sense of self, i.e. on a collection of schema regarding one’s abilities and traits [42]. Accordingly, we here aimed to direct the subjects to reflect on themselves as a person and on their identity. This was realized by issuing a concrete instruction to the subject for self-reflection (e.g. “who am I?”), whereby the subjects could select their own content. As control condition, we instructed subjects to reflect on a personal acquaintance of the same gender. This was to control for the process of reflecting on a personally known person and the related knowledge and memory. Subjects were further requested to reflect on an unknown person who was introduced to them by photograph prior to the functional magnetic resonance imaging (fMRI) session. This controlled for general reflection on a person. In order to intensify reflection, we also presented photographs of the respective persons after the reflection period. Considering the unconventional methodological approach with respect to non-implementation of a behavioural control, we applied very conservative statistics.

Our hypothesis was that the solely mental task of self-reflection would still be associated with brain activity particularly in cortical midline regions such as the medial prefrontal (MPFC) and cingulate cortices, but also in ventrolateral and dorsolateral prefrontal and insular regions and in lateral parietal cortex areas [2,9,25,43]. We predicted different activities for mental self-reflection than for perception of self-related stimuli, thus insinuating self-reflection specific activation. In addition, we were interested in whether or not certain brain regions are specifically associated with self-reflection (refl-self) and self-perception (perc-self) when compared to those areas associated with reflections on other persons (refl-known, refl-unknown) or the perception of photographs of other persons (perc-known, perc-unknown).

## Results

**Figure 1:**
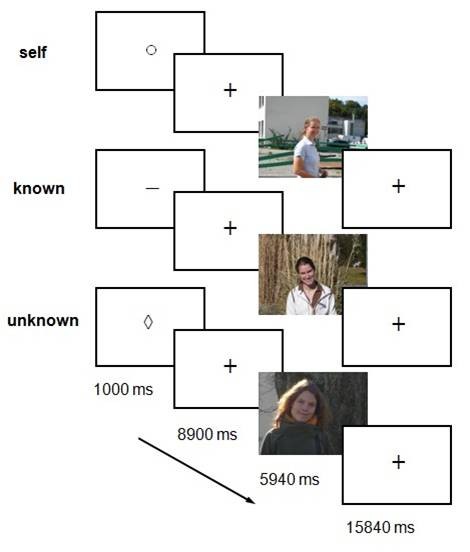
**Brain activity during reflection on oneself.** Analysis of selected regions (yellow circles) with activation during reflecting on oneself, resulting from the conjoined contrast (refl-self > refl-known) & (refl-self > refl-unknown) & (refl-self > perc-self) – (2). The colour-coded maps indicate those areas of the brain with greater t activity during self-reflection than under all other conditions (p < 0.005, Monte Carlo simulation with cluster wise correction p < 0.01). The time courses indicate the mean and standard errors of blood oxygen-level-dependent signal changes under primary conditions. (**A**) DMPFC. (**B**) Ventrolateral prefrontal cortex and insula (VLPFC/Ins. L). (**C**) DLPFC R. (**D**) Posterior cingulate cortex (PCC). All regions have greater activity during the reflection periods than during perception of the photograph. All other significant clusters were also activated under the ‘other’-reflection-conditions.

The primary contrast in the reflection period (1), a conjunction of (refl-self > refl-known) & (refl-self > refl-unknown), revealed those regions of the brain with greater activity under the self-condition compared to these for known-reflection and unknown-reflection, which were among others the bilateral insular and ventrolateral prefrontal cortex (Ins/VLPFC) areas, the ACC, the PCC and the dorsal MPFC (DMPFC; Table 1). The left Ins/VLPFC region, right DLPFC, DMPFC, and PCC showed also more prominent activity in the second conjoined contrast (2), adding (refl-self > perc-self) to contrast (1), under the conditions of self-reflection compared to self-perception (Table 1, Figure 1).

**Table 1:** Analysis of the reflection period

	**Brodmann area**	**Mean X**	**Mean Y**	**Mean Z**	**Cluster size (mm**^**3**^**)**	**t-max**
a) Contrast (refl-self **>** refl-known) & (refl-self **>** refl-unknown)						
Sup. frontal gyrus/DMPFC R, ACC/MCC	6/8/24/32	5	11	37	7132	4.7
Insula/inferior frontal gyrus R	13/45/47	36	23	2	3674	5.1
Insula/inferior frontal gyrus L	13/45/47	-36	21	6	7877	4.7
Middle frontal gyrus/DLPFC R	9	35	29	35	387	3.9
Middle frontal gyrus/DLPFC R	9/10	29	39	24	271	4.0
Middle insular cortex R	13	40	3	2	524	3.5
Midbrain	-	2	-23	0	1361	3.8
Posterior cingulate cortex	23/24	2	-16	33	1281	4.4
Caudate nucleus	-	-17	-6	23	283	3.6
Subcallosal cingulate cortex L	13	-17	11	-10	719	3.9
b) Contrast (refl-self **>** refl-known) & (refl-self **>** refl-unknown) & (refl-self > perc-self)						
Insula/inferior frontal gyrus L (Figure 1A)	13/44	-38	15	8	2495	3.9
Sup. frontal gyrus/DMPFC R (Figure 1B, 2A)	6	9	1	53	1964	4.1
Middle frontal gyrus/DLPFC R (Figure 1 C)	9/10	29	39	24	386	4.0
Posterior cingulate cortex (Figure 1D)	23/24	2	-19	34	636	4.2

Activity during self-reflection significantly larger (random effects analysis, p > 0.005, p < 0.01 Monte Carlo simulation corrected) than in the known and the unknown reflection conditions.

Activation as in a) but also larger in the refl-self than in the perc-self condition.

Abbreviations: R right, L left. DLPFC dorsolateral prefrontal cortex, DMPFC dorsomedial prefrontal cortex, sup superior.

**Figure 2:**
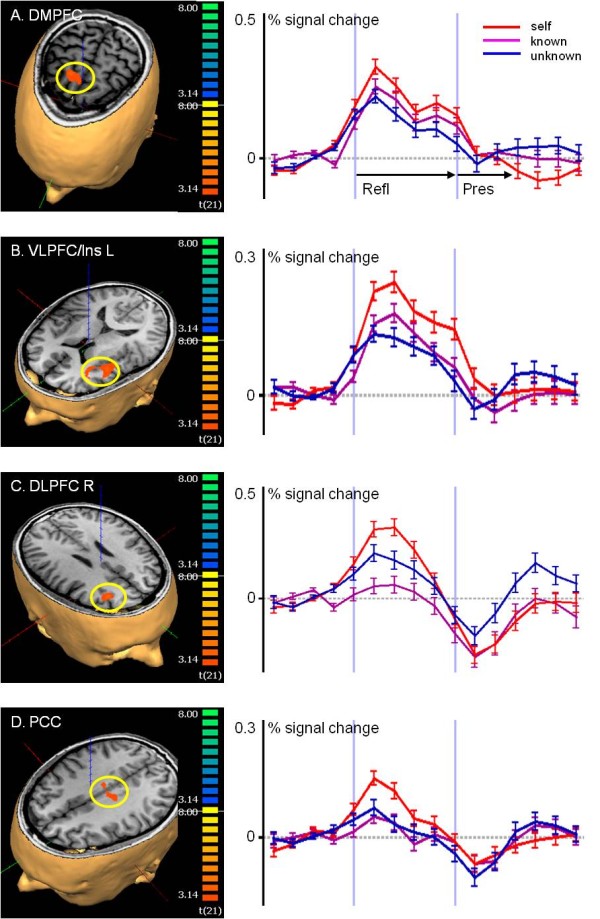
**Beta-weights (beta, y-axis) of the single contrasts.** Given are the beta-weights within selected regions of interest, derived from the contrast analyses in (A + B) reflection-self (r-self) > reflection-known (r-known) and -unknown (r-uk) and perc-self (p-self), and (C + D) perception-self > perception-known (p-known) and -unknown (p-uk). (**A**) DMPFC, (**B**) left ventrolateral prefrontal cortex/insula (Ins L), (**C**) ACC, (**D**) right IPL/SSC.

Assessing the contribution of the single conditions within the resulting clusters, the activity during refl-self was, as expected, most pronounced, although activity was also present during the other conditions of reflection. In contrast, the activity associated with perc-self was not significant compared to the baseline (Figure 2A, B). The DMPFC was significantly active relative to the baseline (t = 9.6) during refl-self, whereas there was no activity during perc-self (t = 0.6; Figure 2A). However, the DMPFC was also active under the conditions of both refl-known (t = 5.9) and refl-unknown (t = 4.5) when compared to the baseline although these activities were significantly less than those for refl-self (refl-self > refl-known: t = 4.4; refl-self > refl-unknown: t = 5.5). Comparable results were obtained for the left insular cortex and adjacent inferior frontal gyrus (Figure 2B). The right middle frontal gyrus corresponding to the dorsolateral prefrontal cortex (DLPFC) was active only in the self-reflection condition (t = 6.2), which was significantly different to the other-conditions where the DLPFC showed no activity or deactivation. The PCC showed equivalent characteristics (refl-self t = 4.8) with no activation or even deactivation in the other conditions.

**Figure 3:**
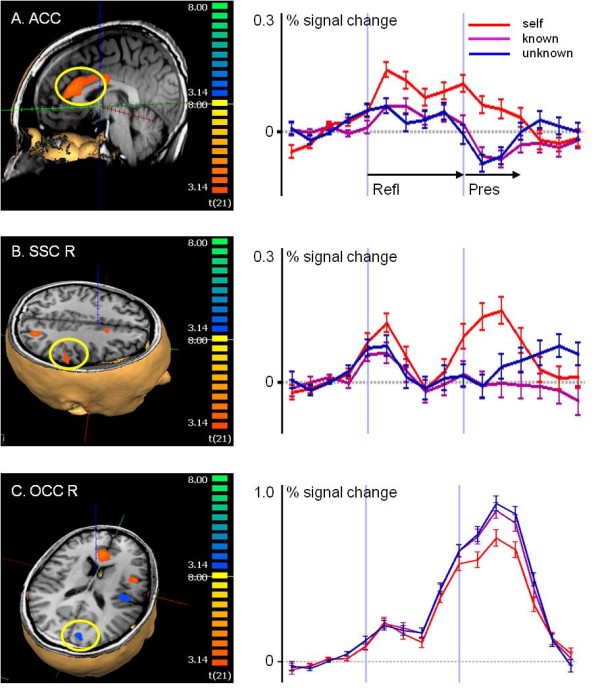
**Brain activity during perception of pictures of oneself.** Analysis of selected regions according to the contrast self-perception with greater activity than under known and unknown perception (p < 0.005, Monte Carlo Simulation with cluster wise correction for multiple comparisons p < 0.01). (**A**) ACC and (**B**) IPL/SSC R, which were also active during self-reflection. (**C**) The occipital visual cortex region was less active during self-perception than under the other conditions.

When examining perception, in particular for the self-specific conjunction (perc-self > perc-known) & (perc-self > perc-unknown), activity analogous to that obtained under the conditions of reflection was detected in the ACC, bilateral insular and ventrolateral prefrontal areas. Additional activity was detected in the right inferior parietal lobe (IPL)/somatosensory cortex (SSC), intraparietal sulcus and bilaterally in the surrounding area of the temporo-occipital junction (Table 2, Figure 3). The ACC activity was self-selective under the conditions of perc-self (t = 2.8; perc-uk t = - 4.0, perc-kn t = - 1.4; perc-self > perc-uk t = 5.7, perc-self > perc-kn t = 5.2) and in part under the conditions of self-reflection (Figure 3A, Figure 2C; refl-self t = 6.4, refl-kn t = 4.3, refl-uk t = 0.7; refl-self > refl-uk t = 6.1, refl-self > refl-kn t = 3.5,).

**Table 2:** Analysis of the picture perception period

	**Brodmann area**	**Mean X**	**Mean Y**	**Mean Z**	**Cluster size mm**^**3**^	**t-max**
Insula/inferior frontal gyrus R	13/47	-36	12	0	833	4.1
Insula/inferior frontal gyrus L	13/47	-36	12	0	833	4.1
Anterior cingulate cortex R (Figure 3A, 2 C)	24/32	2	20	23	5968	5.3
Superior parietal gyrus R	7	25	-61	43	3891	4.6
Inferior parietal lobule R (Figure 3B, 2D)	40/1/2/3	56	-28	35	938	4.0
Temporo-occipital junction R	19/20/37	45	-56	-7	6462	6.4
Occipital cortex R (Figure 3C)	17/18	10	-85	16	490	-3.9

Given are brain regions resulting from the contrast (perc-self > perc-known) & (perc-self > perc-unknown) with activity significantly larger during self-perception and lower than in the known and the unknown condition (random effects analysis, p > 0.005, p < 0.01 Monte Carlo simulation corrected). Clusters with merely deactivations in all conditions in the presentation period were not considered (e.g. bilateral superior/middle temporal gyrus). Abbreviations: R right, L left, med medial. Reported are the mean Talairach coordinates of the respective clusters.

The right IPL/SSC was the only region with significant individual activity attributed to each of the refl-self (t = 2.3) and perc-self (t = 3.2) condition separately and with no significant activity under the conditions of known and unknown (Figure 3B, Figure 2D), and further with differences between the self- and other conditions (refl-self > refl-uk t = 3.3, perc-self > perc-uk t = 4.5, refl-self > refl-kn t = 1.6, perc-self > perc-kn t = 5.0). In occipital regions, particularly in the cuneus, perc-self was associated with less activity than under the other conditions of perception (Table 2, Figure 3C).

## Discussion

### Brain activity associated with self-reflection

The objective of the present study was the identification of brain regions involved in mental self-referential processing during self-reflection upon exclusion of interfering external stimuli, tasks or specific self-referential events. We determined activities in the insular, anterior and posterior cingulate, ventrolateral prefrontal cortex and DMPFC to be involved in the process of self-reference. This emphasizes the role of cortical midline areas [38,44] but also of lateral prefrontal and parietal areas, in self-related processing as self-reflection and converges with the findings of recent meta-analyses on neural correlates in the field of self-referential processes [6,10,11,37], albeit not a complete overlap of all brain regions. The temporoparietal junction (TPJ) was in our study only activated in the picture presentation condition and not in the self-reflection period. This makes sense when considering the proposed role of the TPJ in distinguishing self and other [10] which may be relevant in the perception condition but less during self-reflection. Interestingly, except for in the anterior cingulate cortex region, brain activity was more pronounced when reflecting on oneself than when viewing photographs of oneself. Right DLPFC and PCC even showed self-reflection specific characteristics with stronger activity than the other conditions which showed no activation. The PCC is known to be involved in self-referential processing [11,43], internally directed thoughts [45] and autobiographic memory [46] which were all involved in self-reflection in the current study. In parallel to meta-analytic results on the overlap between self-referential processes and brain regions known to be involved in the default mode network (DMN, [11]) we found prominent activation in the ACC and PCC in the self-reflection condition. This result supports the functional overlap between active and consciously induced self-reflection and self-related mental processes during resting states [11]. Within the frame of our applied conjunctions and the selected statistical threshold, we revealed no relatively stronger activity during self-reflection in ventromedial prefrontal cortex (VMPFC), which has been shown in a comparable PET study on self-reflection [23]. Meta-analyses found consistent results when including studies using PET and fMRI [6,10,11]. However, the long duration of the conditions (100 sec) in the PET study by d’Argembeau et al. could account for this difference in the VMPFC in the two studies. Further, the applied conjunctions lead to restricted results, whereby the single contrast of for instance ‘self’ versus ‘unknown’ reflection was accompanied by VPMPC activity, which however is not reported in detail here due to focusing on the primary question. Another PET study [24], comparing reflections on personality traits and the physical appearance of oneself compared to reflections on a specific famous person resulted in brain activity in dorsolateral and medial prefrontal cortical regions, which is comparable to our results. A recent study attempted to disentangle differential functions of dorso- and ventromedial prefrontal cortex, and detected primarily deactivations in the VMPFC correlating with the degree of self-descriptiveness and increasing activations in DMPFC regions with increasing self-relevance of the given stimuli [47]. It is also possible, that the lack of VMPFC-activation in our study is related to the specific task with no specific given stimuli which had to be judged regarding self-relatedness. Our results and existing literature present evidence for the involvement of insular, cingulate, ventrolateral and dorsolateral and medial prefrontal cortical regions in self-related processing and, as shown in this study, specifically in self-reflection.

### Activations in the known and unknown conditions

Adding to the view that self-related processing activates, in particular, midline prefrontal areas of the brain, our data also revealed activity in these regions during reflection on ‘known’ and ‘unknown’ persons, even although the associated activity was lower than that associated with reflection on oneself. This was indicated by analyzing the various conditions of reflection relative to a baseline and by analyzing local signal changes obtained by fMRI. Our results are in line with previous studies addressing appraisal of oneself and appraisal under the conditions of known and unknown persons [48-50]. The condition ‘unknown’ was also associated with activity in the MPFC and insular regions. The activity under the conditions ‘known’ and ‘unknown’ may well be explained by considering theory of mind [12,51-55]. When reflecting on others, humans engage their mind and activate self-related brain regions for mentalizing others and mental state inference [48,56]. A related explanation is that these brain regions are involved in social cognition with self-related aspects as mirror neuron functions and a co-representation of the self and others which have been reported to involve respective prefrontal midline regions [57-60]. One could argue that the subjects in our study might have dealt with themselves under the ‘other’-conditions. However, our standardized instructions were easily understood by the subjects and they were asked to verbally confirm that they had performed the task precisely as instructed. Further, limitations in the performance of the task would not explain the selective activation in the anterior cingulate and somatosensory cortices under conditions of ‘self’-reflection, which strongly indicate a distinct task performance. Studies using longer phases of reflection involved intensive interviews on the content, self-relatedness and vividness of imaginations, which is necessary to control for interfering thoughts not related to the primary task. However, the conditions we used were, being of less than 10 s duration, relatively short, such that we could initiate the process of self-reflection by letting the subjects reflect on themselves under the question “who am I?” This should have reduced fluctuations in cognition as well as possible mind-wandering.

The primary visual cortex was less active when viewing photographs of oneself than when viewing photographs of other persons. This might be explained by the novelty and unfamiliarity of the unknown person demanding more attention and more cognition and, hence, a correspondingly greater activity in the primary visual cortex [61].

### Self-selective activations

The only regions of the brain that appeared to be of certain selectivity for self-related processing, in the sense of not or hardly exerting activity under the ‘known’ and ‘unknown’ conditions and with significantly stronger activity associated with the self- compared to the ‘known‘and ‘unknown’ conditions, were the ACC and somatosensory-associated parietal areas for the perception period. The term “self-specific” has to be used with caution [62]: in agreement with Gillihan and Farah, we did not claim to find structures solely involved in self-referential processes but rather to analyse associations between brain regions and self-reference which is also reflected in the overlap of self and familiar other related brain regions in two meta-analyses [10,11]. With regard to the objections of Gillihan and Farah [62], our study tried to circumvent some of the constraints of previous studies such as possible interferences of verbal or visual stimulus material used for referential processes with the aspect of self-reference itself. Our study adds to the existing literature by using a more naturalistic approach similar to everyday self-referential thinking without direction onto specific stimuli and contents.

The ACC was further more strongly stronger activated during self-reflection compared to ‘known’ and unknown’ reflection, whenever the ‘known’ reflection exerted slight activity. The ACC is known to be associated with conflict monitoring and to detect discrepancies between the actual and the desired state [63,64]. Functionally, cingulate regions are known to mediate integration and evaluation of emotional, motivational and cognitive information, and to modulate attention [65,66] with direct connections to the amygdala, thalamus, prefrontal and insular areas and to the parietal lobe [67]. Thus, within a regulatory network, the ACC may have the function of assessing whether signals or stimuli have a relation to or relevance for the person as was proposed by van der Meer et al. [6]. However, following the logic of the ACC functioning as a discrepancy detector, why should it be active when one reflects on oneself? In this study, we instructed the subjects to reflect about whom they were and how they were. This may have initiated autobiographical considerations on the current state and the intended goals in life. The task may thus have induced critical self-reflection and have made the subject aware of discrepancies between his/her actual state and desired state. This self-reflection would, of course, be reduced, or perhaps not occur at all, when reflecting on other persons. A recent study [14] investigated brain activity in subjects viewing photographs of their own face or body in comparison to the activity when viewing photographs either of a close colleague or of scrambled images. This also was associated with activation of the ACC, the right anterior insula and the adjacent inferior frontal gyrus. The authors proposed that the insula and the ACC could provide an abstract representation of the self that might participate in maintaining the sense of self. Similarly, another study addressing the relation of trait adjectives to the own person and an acquaintance identified insular and anterior cingulate cortex activation to differentiate between self and other reference with a self-specificity for insular activity [25].

An electroencephalography study [12] reported reflective self-reference in contrast to other-reference to activate more DMPFC regions and the right anterior insula. Pre-reflective self-reference was accompanied by left insular and dorsal and ventral prefrontal activities. This is consistent with our findings assuming that we did not differentiate between pre-reflective aspects and reflective self-reference. Pre-reflective self, or ‘minimal’ self, was defined as ‘consciousness of oneself as an immediate subject of experience, not extended in time’ [68]. A contribution of the ‘minimal’ self as a basic function may generally be present in self-referential processing and, particularly, during self-reflection. The task we used in this study was supposed to involve both reflective and pre-reflective processes, which is reflected by our findings on brain activation.

The other brain region specifically activated under the conditions of ‘self’ was the IPL comprising the region ‘PF’ [69], the ventral intraparietal cortex corresponding to the IPL and parts of the postcentral bank hosting the SSC. These regions are strongly associated with somatosensory processing, especially the anterior bank of the parietal lobule, and also integrate visual and auditory information [69-71]. They may thus form a higher-order perceptional representation of the self as a result of integration of multimodal sensory information. In this study, these areas were active in the mental condition, supporting a concept that they may also provide for a self-representation when mentally directing attention to oneself or when a self-image is required for reflection purposes or for self-identification.

We confirm earlier findings that the mentioned areas of the brain are associated with self-reflection, whereby our strict and conservative approach has to be considered with regard to areas not reported, such as the anterior medial prefrontal cortex regions, the temporal cortex and the subcortical regions as upper brainstem [2,9,43].

Results on self-reflection processes may have implications for psychiatric disorders which have self-related psychopathologies [1,72-75]. There is evidence that, for example, depression and social anxiety disorder are both associated with a heightened cognitive, mostly ruminative self-focus, pointing to dysfunctional activation of brain areas involved in self-related processing [1,76,77]. A prominent part of self-related processing in affective disorders such as depression occurs without external stimuli, which is comparable to the conditions used in the present study. A task for the future will be to assess the involved regions of the brain for possible alterations in self-processing, such as, for example, dysfunctional attractors in depression.

## Conclusion

Our findings underline the concept of a primary involvement of medial and also lateral prefrontal, parietal and insular regions in self-related processing, demonstrated here with an experimental task abstaining from externally oriented behavioural requirements. These areas, as the DMPFC, were also active while reflecting on others, thus putting a certain self-selectivity into perspective. Specificity for self-related activation was shown only in the ACC and in regions of the inferior parietal cortex associated with somatosensation. From a methodological viewpoint, we emphasize here the expected and plausible association of these areas with a mental self-related condition in the absence of external stimuli and without required behavioural responses. This underlines the possibility of using such an approach for investigating self-related neural processing. The reported areas may be considered when investigating disturbed self-related processing in psychiatric disorders.

## Methods

### Participants

Twenty-five healthy subjects (aged 23-41 years, 17 females, all right-handed according to a handedness questionnaire [78]) were contacted by direct address or mailing lists. Subjects were screened for exclusion criteria such as prior and current neurological or psychiatric illnesses, the use of medication or other psychotropic substances (excluding contraceptives), pregnancy, excessive consummation of alcohol, cigarettes and caffeine, and the general contraindications for fMRI. The study was approved by the ethics committee of the canton of Zürich (project nr. E 62/2007) and conducted in compliance with the Declaration of Helsinki. All subjects provided their written informed consent. After scanning, subjects completed a post-experimental non-structured interview on their performance of the task. Two subjects reported sleepiness and lack of concentration during fMRI and one subject showed several movement artefacts (sudden head movements of more than 3 mm). The data for these three subjects were, therefore, excluded from the analysis. The other subjects confirmed that they had been able to follow the instructions.

**Figure 4:**
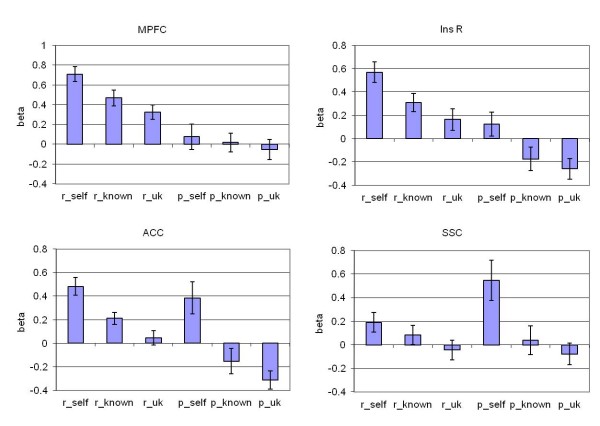
**Schematic summary of the paradigm.** The paradigm comprises a cued reflection period followed by perception of a photograph to the cued person for the indicated periods of time. For each condition, the same standardized set of photographs was shown in a random order. The subjects were instructed to reflect on the cued person in the ‘reflection’ period and to view the photograph during the ‘perception’ period (photos reprinted here with explicit consent of the shown subjects).

Prior to scanning photographs of each subject (‘self’) or for each subject of a person known to him/her (‘known’; work or study colleague but not a close friend or a relative since intense emotional relationships would interfere with the results), or of a person of the same sex and not known by the subjects (‘unknown’) were obtained. The photographs were taken within the grounds of the Psychiatric University Hospital, Zurich. Situations, positions (portrait in relation to whole body), background and perspectives were standardized in a script (for examples of the photographs, see Figure 4). Subjects were instructed to display relaxed and neutral facial expressions and body positions, in particular not to express any explicit emotions, although a normal friendly ‘photo-smile’ was permitted. Clothing was neutral and casual. Photographs presented to each subject of always the same known person and always the same unknown person and were matched for gender and approximate age. The subjects were shown photographs of the known person and the unknown person prior to scanning.

### Experimental design

The experimental task comprised a cue indicating the trial conditions, a period for mental reflection without external stimuli, then a period for perception of the relevant photograph (Figure 4). To investigate only the process of self-referential reflection, the subjects were instructed to generate self-reflective cognitions in the absence of external stimulation other than the short abstract cue (1 s) indicating the beginning of the trial and the person concerned. The periods for reflection lasted less than 10 s, thus primarily representing the induction and early phase of mental reflective processes. Photographs of themselves were introduced to each subject in order to compare brain activities under conditions of reflexion and perception. Self-reflection and perception were also controlled under conditions of reflection on and perception of either a known or an unknown person. The ‘known’ condition consisted of a period to reflect on a person who had a distant relationship to the subject’s self, such as a colleague or an acquaintance. The ‘unknown’ condition, serving as a control, consisted of a period for reflection on an unknown person, followed by the viewing of a photograph of this unknown person.

Each trial period was initiated by a cue as instruction and terminated by the disappearance of the photographs (Figure 4). The subjects were instructed and trained before scanning as follows: “The cue indicates a person who will later appear in a photograph. In the period that follows, reflect about that person, who and how she/he is or might be, for instance ‘who am I, how am I as a person’ or ‘who and how is she/he’. When the photograph is presented, just look at the photo”. The instructing cues for self: ○, known: or unknown: ◊, were presented for 1 s. They were graphically abstract and without any intrinsic meaning. Including the cue, the reflection conditions lasted 9900 ms, equivalent to 5 TRs (repetition time for the fMRI volumes). The photographs were presented for 5940 ms, equivalent to 3 TRs. Then, a baseline period of 8 TRs followed until the next trial was started. In total, each subject performed each condition 12 times in a randomized order. The task was programmed with PresentationTM, Neurobehavioral Systems, USA. After scanning, subjects were interviewed on their ability to perform the task; all included subjects were completely able and fulfilled this criterion. The main information reported for self-reference were individual facts (such as name, age, employment and family status) as well as autobiographical and future-oriented pieces of information (such as “I was born in …”, “I want to be …”, “I am …”). The subjects were not questioned on the personal content of their reflection in order to avoid self-confrontation or the disclosure of private thoughts.

When designing the experimental task, we purposely did not use external stimuli or an external relation as implemented motor responses, nor did we use cognitive or behavioural task for instance requiring decision making. This was to avoid any interference with the self-reflection process, and, hence, to enable us to focus on the mental component of the reflection task. Such, the solely mental self-reference is meant in the sense of an absence of possibly interrupting external stimuli. One might argue that a behavioural response for task performance or a control for attentiveness during the task is lacking, but such an additional task would have directed the attention of the subjects to the performance of this controlling task and away from the performance of the primary task. In order to control for subject attentiveness and cooperation, the direct task-related activity in each subject’s primary visual cortex was controlled to assure that at least visual perception was functional since, for example, closing of the eyes would have resulted in missing or low activity in the visual cortex at least during the perception of the pictures.

### Image acquisition

Imaging was performed using a 3.0 T GE SignaTM HD Scanner (GE Medical Systems, Milwaukee, USA, 8-channel head coil). Echo-planar imaging (EPI) was performed for fMRI (TR/TE 1980/32 ms, 22 sequential axial slices, whole brain, 3.5 mm slice thickness, 1 mm gap, resulting voxel size 3.125 × 3.125 × 4.5 mm, matrix 64 × 64 voxel, 200 mm field of view, 70° flip angle). 468 volumes were obtained per subject, 16 volumes per trial. The first 4 volumes were discarded to allow for T2 equilibration effects. High-resolution 3-D T1-weighted anatomical volumes were acquired (TR/TE 9.9/2.9 ms; matrix size 256 × 256 mm; 1 × 1 × 1 mm resolution, axial orientation) for co-registration with the functional data. T2-weighted images in parallel to the EPI sequence were acquired to exclude possible T2-sensitive abnormalities. The stimuli were presented via digital video goggles (Resonance Technologies, Northridge, CA).

### Data analysis and statistics

FMRI data were analyzed using BrainVoyagerTM QX 1.10.1 (Brain Innovation, Maastricht, The Netherlands [79]). Pre-processing of the functional scans included motion correction, slice scan time correction, temporal high-pass filtering and removal of linear trends. Functional images were superimposed on the anatomical images and incorporated into 3-D data sets. The individual 3-D data sets were transformed into Talairach space [80], with a resulting voxel size of 3 × 3 × 3 mm, then spatially smoothed with an 8-mm Gaussian kernel for subsequent group analysis. Six predictors, representing the three conditions each of reflection (refl) and perception (perc, refl-self, perc-self, refl-known, perc-known, refl-unknown, perc-unknown) were used to build the design matrix. Single trials with fMRI signal artefacts of more than threefold mean signal change amplitude (e.g. due to head movements) were eliminated. The trial periods were modelled as epochs using a two-gamma hemodynamic response function adapted to the applied period duration provided by BrainVoyager.

The fMRI data analysis, based on the general linear model, comprised the following steps: First, fixed effects analyses were calculated separately for each subject under the conditions of reflection and perception for ‘self’, ‘known’, ‘unknown’, each resulting in summary images. The summary images were subjected to second-level random effects group analyses, also using conjunctions (&). Three-dimensional statistical parametric maps were calculated for the group analysis with separate subject predictor using random effects analyses, whereby we controlled for and avoided the minimum-t problem [81] by building masks of the single contrasts and verifying that the resulting clusters from the conjunctions are within these clusters and such represent a logical AND-condition. The statistical threshold for reporting results was set to p < 0.005 and to a cluster-wise level of p < 0.01 (considering the Talairach voxels of 3 × 3 × 3) mm after correction for multiple comparisons by application of the respective cluster thresholds according to a Monte Carlo simulation [79].

We were interested in brain activity during self-reflection and in this context for “self-specific” activation meaning here significantly more activation in the self-conditions than in the other conditions with these not showing activation. Therefore, the main contrasts were:

Self-specific conjoined comparison in the reflection period:

(refl-self > refl-known) & (refl-self > refl-unknown)

as primary outcome measure.

To identify areas with greater activity during self-reflection than during self-perception, the additional conjunction with the contrast (refl-self > perc-self) resulting in the contrast:

(refl-self > refl-known) & (refl-self > refl-unknown) & (refl-self > perc-self)

as secondary outcome measure.

To differentiate the contributions of each separate condition to the clusters resulting from the conjunction analyses (and corresponding to the main effect), we conducted a post-hoc analysis in which an additional random-effects analysis within the resulting clusters was performed to determine the effects of each single condition on brain activity. Self-selectivity in our context was defined as significant activity in a region during reflection or perception only under the ‘self’-condition and not under the conditions ‘known’ or ‘unknown’.

For reasons of comparison with previous studies addressing self-selectivity during the perception of pictures of the own and other persons, the conjunction (perc-self > perc-known) & (perc-self > perc-unknown) was analyzed. In the resulting clusters with self-selective activation characteristics, the effects of the individual conditions were investigated in a post-hoc random-effects analysis as described above.

## Competing interests

All authors declare that there are no competing interests.

## Authors’ contributions

The study was conceived and designed by UH, LJ and AB. MRI measurements and statistical analyses were carried out by AB, TK, CS. The data were interpreted by UH, AB, CS and TK. The manuscript was drafted by UH and AB, and all authors revised it critically for important intellectual content. All authors approved the final manuscript.
